# Feasibility of IVIM parameters from diffusion-weighted imaging at 11.7T MRI for detecting ischemic changes in common carotid artery occlusion rats

**DOI:** 10.1038/s41598-020-65310-8

**Published:** 2020-05-21

**Authors:** Shunrou Fujiwara, Yuki Mori, Daniela Martinez de la Mora, Yosuke Akamatsu, Kenji Yoshida, Yuji Shibata, Tomoyuki Masuda, Kuniaki Ogasawara, Yoshichika Yoshioka

**Affiliations:** 1 Department of Neurosurgery, Iwate Medical University, 1-1-1 Idaidori, Yahaba, Iwate 028-3694 Japan; 2 Graduate School of Frontier Science, Osaka University, 3-1 Yamadaoka, Suita, Osaka 565-0871 Japan; 3 Center for Translational Neuromedicine, University of Copenhagen, Blegdamsvej 3B, 2200 Copenhagen N, Denmark; 4 Department of Pathology, Iwate Medical University, 1-1-1 Idaidori, Yahaba, Iwate 028-3694 Japan; 5 Center for Information and Neural Networks (CiNet), NICT and Osaka University, 3-1 Yamadaoka, Suita, Osaka 565-0871 Japan

**Keywords:** Image processing, Preclinical research, Software

## Abstract

This study aimed to investigate whether intravoxel incoherent motion (IVIM) parameters can identify ischemic changes in the rat cerebral cortex using a preclinical ultra-high-field 11.7 Tesla magnetic resonance imaging (11.7TMRI) scanner. In nine female Wistar rats (eight weeks old), diffusion-weighted imaging (DWI) for IVIM analysis was successfully performed before (Pre) and after unilateral (UCCAO) and bilateral (BCCAO) common carotid artery occlusion. From the acquired DWI signals averaged in six regions of interest (ROI) placed on the cortex, volume fraction of perfusion compartment (F), pseudo diffusion coefficient (D*), F × D* and apparent diffusion coefficient (ADC) were determined as IVIM parameters in the following three DWI signal models: the bi-exponential, kurtosis, and tri-exponential model. For a subgroup analysis, four rats that survived two weeks after BCCAO were assigned to the long survival (LS) group, whereas the non-LS group consisted of the remaining five animals. Each IVIM parameter change among three phases (Pre, UCCAO and BCCAO) was statistically examined in each ROI. Then, the change in each rat group was also examined for subgroup analysis. All three models were able to identify cerebral ischemic change and damage as IVIM parameter change among three phases. Furthermore, the kurtosis model could identify the parameter changes in more regions than the other two models. In the subgroup analysis with the kurtosis model, ADC in non-LS group significantly decreased between UCCAO and BCCAO but not in LS group. IVIM parameters at 11.7TMRI may help us to detect the subtle ischemic change; in particular, with the kurtosis model.

## Introduction

Preclinical ultra-high-field magnetic resonance imaging (MRI) with static magnetic fields of more than 7 Tesla (T) has identified even subtle microstructural morphological and functional changes in biological tissues using diffusion-weighted imaging (DWI)^1–4^. Intravoxel incoherent motion (IVIM) is a DWI concept, and it has been performed to non-invasively and simultaneously assess perfusion and diffusion from DWI datasets obtained in a one-time scan with multiple b-values^5–13^. In the period from the 1980s to nowadays, IVIM analysis has been mainly used to assess animal models of liver fibrosis or brain tumors, as well as patients with cancer in various regions of the body^7,11,12,14–20^. With the further development of the magnetic field strength, recent studies have shown that IVIM can detect cerebral perfusion changes not only in patients with infarcts due to severe ischemic strokes or vasospasms after aneurysm rupture but even in healthy subjects in hyper- or hypocapnic states^9,10,21–23^. For IVIM analysis, various DWI signal models have been proposed to assess water diffusion features in different views such as diffusivities based on free (Gaussian) and restricted water molecules or fast and slow diffusivities in multiple compartments of the tissue^7,10^; however, it remains unknown which DWI model of IVIM parameter estimation can appropriately identify subtle changes in ischemic states, especially at early stages without the presence of infarcts seen in chronic ischemia.

Ischemia can be accomplished in animal models by vessel occlusion. This has been proposed to determine longitudinally the extent of brain damage caused by chronic cerebral hypoperfusion^24–27^. Among the established models, the common carotid artery occlusion (CCAO) model has the advantage of consistently inducing cerebral hemodynamic changes and postischemic neural degeneration without the possibility of surgical injuries to the cerebral cortex. However, longitudinal studies using this model often include a large number of animals because this model has a high mortality. A staged ligation, in which the ligation of the second carotid artery was performed 2–7 days after the first one, has improved the mortality^2,26,27^; however, it has been unclear how cerebral hemodynamics and cortical damages change in rats after CCAO.

Using ultra-high-field 11.7 T magnetic resonance imaging (11.7TMRI), we investigated in this study which DWI model of IVIM parameters sufficiently identifies different ischemic states, how the cerebral hemodynamics changes in the CCAO model, and how the rat cortex is damaged in this animal model.

## Results

**Figure 1:**
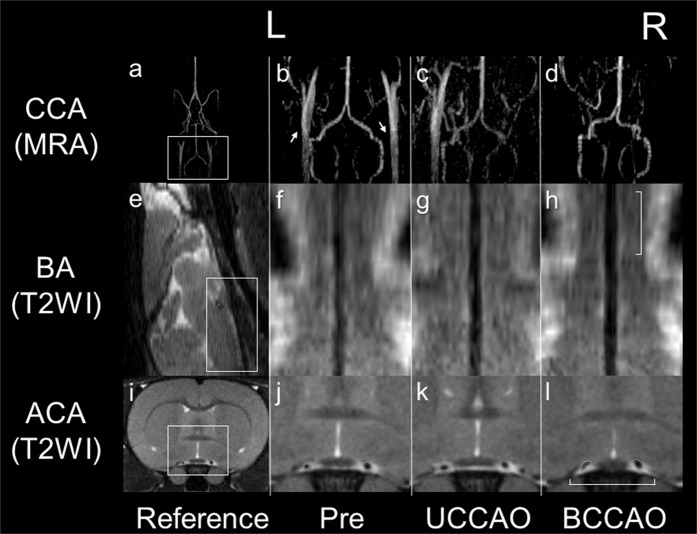
Morphological changes in blood vessels on 11.7 Tesla magnetic resonance (MR) images before and after common carotid artery occlusion (CCAO). The squares in (**a,e,i**) indicate the references for (**b–d,f–h,j–l**), respectively. On MR angiography (MRA) images before CCAO (Pre), the CCAs can be identified bilaterally (white arrows in **b**). L and R are the left and right sides, respectively. After unilateral occlusion of the right CCA (UCCAO), only the left CCA is visualized (**c**), and after bilateral CCAO (BCCAO), the CCAs cannot be identified (**d**). The diameters of the basilar artery (BA) measured on curved planar reformation T2-weighted images (T2WI) increase over the occlusion period (**f–h**). The cross-sectional area of the anterior cerebral artery (ACA) at the section showing the anterior commissure increases slightly in the UCCAO (k) and BCCAO (**l**) images compared with the image before the occlusion (**j**). Scale bars, 5 mm.

Bilateral CCAO (BCCAO) was successfully performed in 10 of 11 rats; one rat died immediately after BCCAO surgery. MRI was performed in these 10 rats before the ligation (Pre), after ligation of the right common carotid artery (unilateral CCAO; UCCAO), and after subsequent ligation of the left common carotid artery (BCCAO). A typical example is shown on Fig. 1. Since one rat was excluded from further analyses because of extensive image deterioration, 9 of 11 rats were used for the analysis. A total of 4 of these 9 rats (44%) survived for two weeks and were assigned to the long survival (LS) group, whereas the remaining 5 rats (56%) were assigned to the non-LS group (survival after BCCAO: range 1–4 days, mean 1.6 ± 1.2 days).

### Morphological changes in the cerebral arteries due to ischemia in all rats

**Figure 2:**
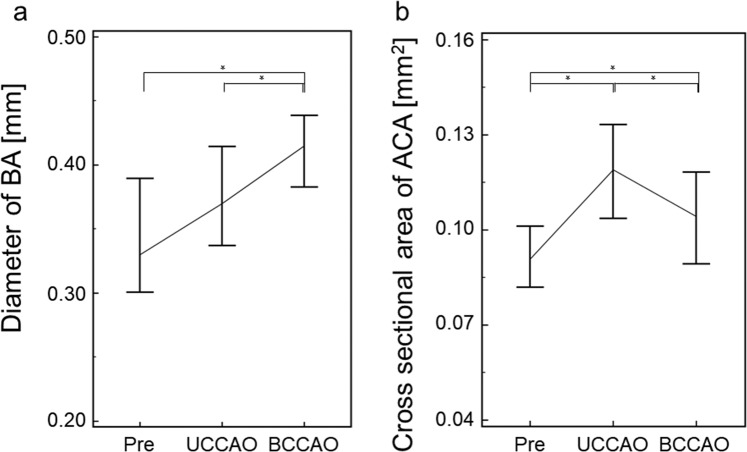
Quantitative assessment for changes in diameters of the basilar artery (BA) (**a**) and in cross-sectional areas of the anterior cerebral artery (ACA) (**b**) before (Pre) and after unilateral (UCCAO) and bilateral (BCCAO) common carotid artery occlusions (*significantly different at the level p < 0.05).

Cerebrovascular morphological changes were observed on T2-weighted images obtained using a rapid acquisition with relaxation enhancement (RARE) sequence. These measurements were performed two times by an author and showed good agreements for the parameters lumen diameter (Dia__BA_) of the basilar artery (BA) and bilateral lumen cross-sectional areas (Area__ACA_) of the anterior cerebral arteries (ACA) as demonstrated by high intraclass correlation coefficients (both 0.92). Both Dia__BA_ (n = 9, median, interquartile range (IQR) [mm]: Pre, 0.330, 0.082; UCCAO, 0.370, 0.065; BCCAO, 0.415, 0.038; p = 0.00294, Friedman test) and bilaterally averaged Area__ACA_ (n = 9, median, IQR [×10^−2^ mm^2^]: Pre, 9.23, 2.46; UCCAO, 11.5, 3.48; BCCAO, 11.1, 2.01; p < 0.00001, Friedman test) were significantly higher for the UCCAO and the BCCAO phase compared to the Pre phase (both p < 0.05, Conover *post hoc* test; Fig. 2). No statistically significant difference was observed in the parameter Dia__BA_ between Pre and UCCAO. However, the value of the parameter Area__ACA_ was in the BCCAO phase lower compared to the UCCAO phase (p < 0.05, Conover *post hoc* test; Fig. 2).

### IVIM parameter changes in all rats

Before (Pre) and after (UCCAO and BCCAO) the occlusion, IVIM parameters were successfully estimated in all rats using the following three typical DWI models: the bi-exponential (Bi), kurtosis (Kur), and tri-exponential (Tri) model. For each model, the IVIM parameter dataset at each phase (Pre, UCCAO, and BCCAO) was determined using an averaged DWI signal change obtained in each region of interest (ROI) placed at the internal, medial, or lateral part of the right or left cortex. Simultaneously, Akaike’s information coefficient (AIC)^10,28,29^, which is an index for signal model functions to assess their goodness of fit, was calculated. This means that IVIM parameters for every signal model were calculated in 162 ROIs (six ROIs × nine rats × three phases). ROI placement and evaluation of the parameters were automatically performed by a custom-made software (calculation time, 176 second per rat for all three models). Finally, each of four IVIM parameters (F, D*, FD*, and ADC) obtained in each of 54 ROIs was statistically compared among the three occlusion phases using the Friedman test for the three examined models (all medians, 25^th^ and 75^th^ percentiles and significances shown in Supplement).

#### Bi-exponential model

**Figure 3:**
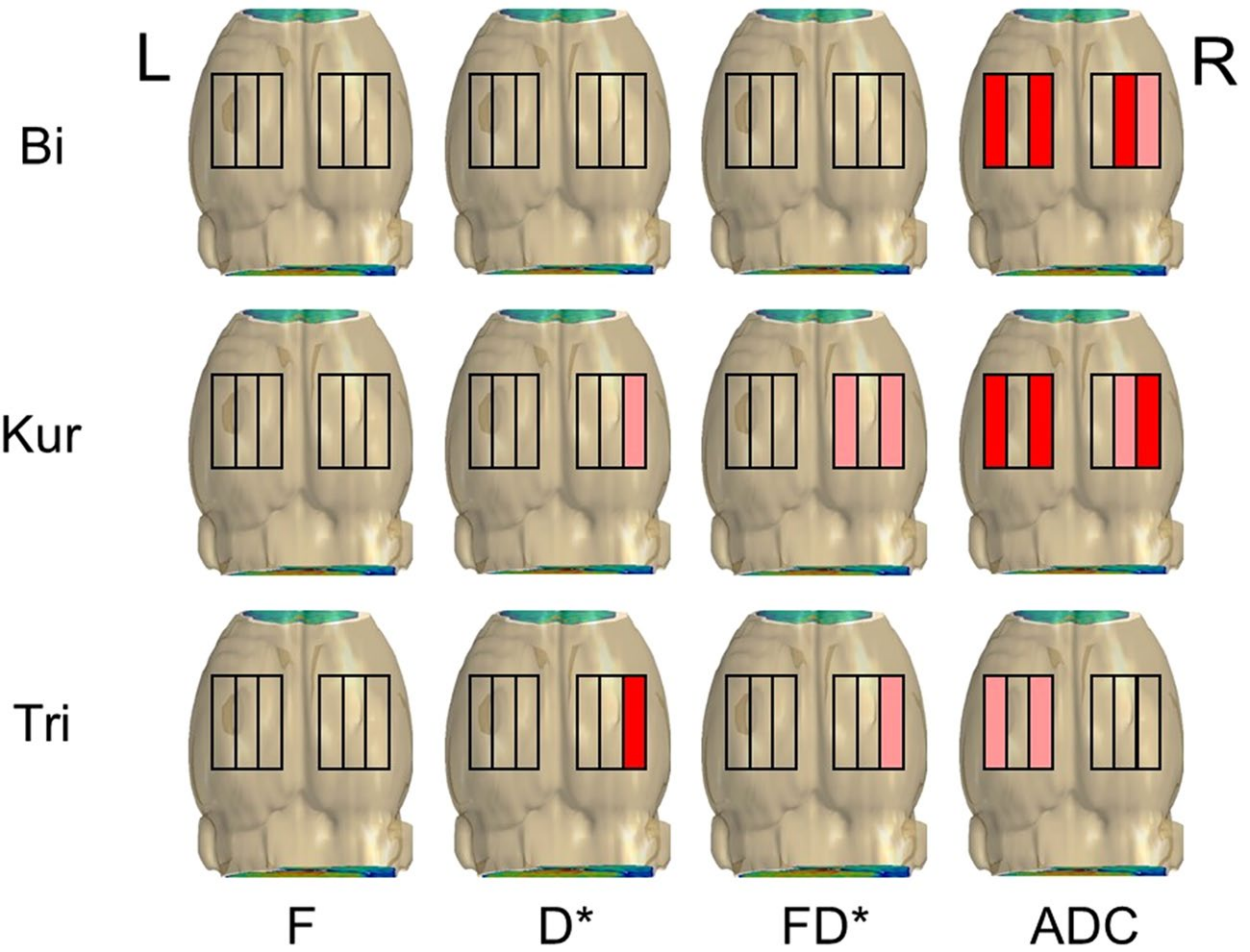
Cortical regions to place regions of interest (ROI) (three black squares: internal, medial, lateral) on both right and left sides in the rat cortex for estimating intravoxel incoherent motion parameters. Significant change among three occlusion phases was observed in each colored regions (pink, p < 0.05; red, p < 0.01; Friedman test).

The parameter ADC_B_ was significantly different among all three phases at the internal and lateral parts of the left cortex, and the medial and lateral parts of the right cortex (n = 9, p < 0.05 at each part, Friedman test, colored parts in the upper column on Fig. 3), and it was significantly lower in BCCAO than in Pre and UCCAO at all significant parts (both p < 0.05, Conover *post hoc* test after Friedman test). By contrast, the remaining IVIM parameters F_B_, D*_B_, and F_B_D*_B_ at all parts of both cortices showed no significant differences among the three examined phases.

#### Kurtosis model

In the kurtosis model, the parameter ADC_K_ at the internal and lateral parts of the left cortex exhibited significant differences among the three occlusion phases (n = 9; p < 0.05 at each part, Friedman test, colored parts in the middle column on Fig. 3). Then, D*_K_, F_K_D*_K_, and ADC_K_ at the lateral part of the right cortex showed significant differences among the three occlusion phases (n = 9; p < 0.05 at each part, Friedman test, colored parts in the middle column on Fig. 3). In addition, F_K_D*_K_ at the internal part and ADC_K_ at the medial part of the right cortex also showed the significance among all phases (n = 9; p < 0.05 at each part, Friedman test, colored parts in the middle column on Fig. 3). On the other hand, F_K_ was not significantly different among all three phases at all parts of both cortices. ADC_K_ at the internal and lateral parts of the left cortex were significantly lower in BCCAO compared to Pre and UCCAO (both p < 0.05, Conover *post hoc* test after Friedman test). D*_K_, F_K_D*_K_, and ADC_K_ at the lateral part of the right cortex were also lower in BCCAO than those in the other two phases (both p < 0.05, Conover *post hoc* test after Friedman test). F_K_D*_K_ at the internal part and ADC_K_ at the medial part of the right cortex were lower in BCCAO compared to Pre (p < 0.05, Conover *post hoc* test after Friedman test).

#### Tri-exponential model

In this model, D*_T_, F_T_D*_T_ at the lateral part of the right cortex and ADC_T_ at the internal and lateral parts of the left cortex showed statistically significant differences among all three phases (n = 9; p < 0.05 at each part, Friedman test, colored parts in the lower column on Fig. 3), whereas F_K_ was not significantly different at all parts of both cortices. The parameters D*_T_, F_T_D*_T_, at the lateral part of the right cortex were significantly lower in BCCAO than in UCCAO (both p < 0.05, Conover *post hoc* test after Friedman test). ADC_T_ at the internal part of the left cortex was lower in BCCAO than in Pre and UCCAO (both p < 0.05, Conover *post hoc* test after Friedman test) and ADC_T_ at the lateral part of the left cortex was lower in BCCAO than in Pre (p < 0.05, Conover *post hoc* test after Friedman test).

Finally, number of the regions showing the significance in IVIM parameter changes for Pre, UCCAO, and BCCAO were the largest when using Kur model (four regions in Bi and Tri and seven regions in Kur; Fig. 3). The AIC comparison revealed significant differences among the three examined models (p < 0.0001, repeated measures analysis of variance) and the mean AIC value was for the Kur model significantly smaller compared to the Bi and the Tri models (mean ± standard error: Bi: 71.8 ± 0.38; Kur: 71.3 ± 0.52; Tri: 88.7 ± 0.54; Bi v.s. Kur, p < 0.0246; Bi v.s. Tri, p < 0.0001; Kur v.s. Tri, p < 0.0001; all pairwise comparisons with Bonferroni correction). An AIC value below 72 meant that the estimated model curve sufficiently approximated the experimental DWI signals.

### Subgroup analysis

#### Comparison of the internal carotid artery (ICA) flow between LS and non-LS groups

**Figure 4:**
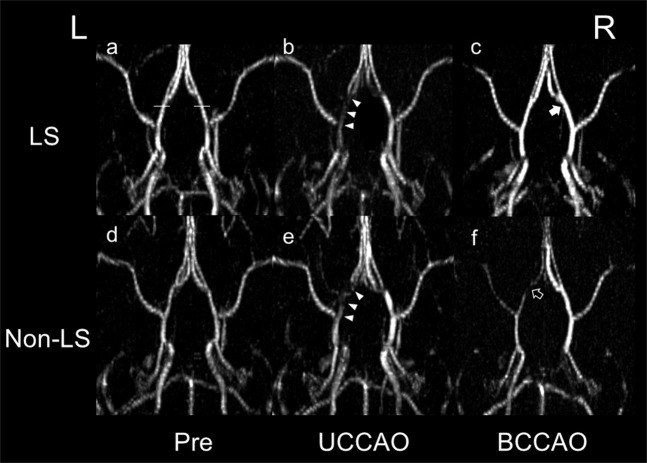
Differences between the long survival (LS) and non-LS groups in magnetic resonance angiography. L and R are the left and right sides, respectively. After unilateral common carotid artery occlusion (UCCAO), rats in both groups commonly show the mild signal loss indicating a blood flow reduction in the internal carotid artery in the circle of Willis contralateral to the ligation (**b,e**, arrowheads) compared with the presentation before the occlusion (Pre, **a,d**). This reduction tends to be reversed after bilateral common carotid artery occlusion (BCCAO) in the LS group (**c**) accompanying the signal increase indicating the compensation by the ICA enlargement (**c**, white arrow). On the other hand, the reduction is severely progressed in the non-LS group (**f**, black arrow). White horizontal lines in (**a**) indicate the reference positions to measure the cross-sectional areas of the ICA flow in the circle of Willis on an MRA.

On magnetic resonance angiography (MRA) in the UCCAO phase, the signal on the right side ipsilateral to the first CCA ligation (the first side) tended to increase in both groups compared to that in the Pre phase. Interestingly, all rats showed in the UCCAO phase a mild signal loss indicating a flow reduction in the ICA at the portion of the circle of Willis on the left side contralateral to the first CCA ligation (the second side) (Fig. 4b,e, white arrowheads). On the other hand, in the BCCAO phase, the signal on the first side in LS group further increased comparing with that in the UCCAO (Fig. 4c, white arrow); however, the signal in non-LS group mildly decreased (Fig. 4f). Then, the ICA flow signal on the second side further decreased only in the non-LS group (Fig. 4f, black arrow), whereas the signal tended to recover in the LS group (Fig. 4c).

For the quantitative analysis, the cross-sectional area of the ICA flow on MRA (Fig. 4a, horizontal white lines indicating the position of the sections) were measured by high intraclass correlation coefficients (0.90). In LS group, the quantitative assessment clearly showed that the cross-sectional area of the flow on the first side significantly enlarged in the BCCAO phase compared to Pre and UCCAO (n = 5, median, IQR [×10^−2^ mm^2^]: Pre, 9.94, 1.73; UCCAO, 12.8, 1.35; BCCAO, 14.8, 1.19; p = 0.0065, Friedman test; p < 0.05, Conover *post hoc* test). Non-LS group showed no significant difference in the ICA flow area on the first side among three phases (n = 4, Pre, 9.84, 6.40; UCCAO, 14.2, 6.57; BCCAO, 10.4, 2.29; p = 0.1680, Friedman test). On the second side, the ICA flow area in non-LS group significantly reduced in BCCAO phase compared to UCCAO (n = 5, Pre, 10.4, 0.923; UCCAO, 11.4, 1.26; BCCAO, 5.46, 3.78; p = 0.0531, Friedman test; p < 0.05, Conover *post hoc* test). The area on the second side in LS group showed no significance among three phases (n = 4, Pre, 10.3, 1.89; UCCAO, 11.9, 1.67; BCCAO, 8.52, 3.32; p = 0.1780, Friedman test).

#### Comparison of IVIM parameter changes in LS and non-LS groups

For confirming differences of damage to the cortex between two rat groups, each IVIM parameter change among the three occlusion phases were statistically examined in each rat group at seven regions showing the significance in all rat analysis with Kur model (D*_K_ at the lateral part, F_K_D*_K_ at the internal and lateral parts, and ADC_K_ at the medial and lateral parts of the cortex on the right side ipsilateral to the first ligation, and ADC_K_ at the internal and lateral parts of the cortex on the left side contralateral to the first ligation, colored parts in the middle column on Fig. 3).

At the lateral part of the cortex on the first side, D*_K_ (n = 5, median, IQR [×10^−3^ mm^2^]: Pre, 27.0, 41.5; UCCAO, 38.0, 80.4; BCCAO, 22.7, 60.4; p = 0.5997, Friedman test), F_K_D*_K_ (median, IQR [×10^−4^ mm^2^]: Pre, 23.9, 33.4; UCCAO, 32.9, 38.7; BCCAO, 14.4, 16.2; p = 0.4979) and ADC_K_ (Pre, 7.33, 0.37; UCCAO, 7.83, 0.84; BCCAO, 7.17, 0.42; p = 0.4979) in the non-LS group, and D*_K_ (n = 4, median, IQR [×10^−3^ mm^2^]: Pre, 30.2, 26.7; UCCAO, 24.0, 32.2; BCCAO, 19.7, 24.3; p = 0.4807, Friedman test), F_K_D*_K_ (median, IQR [×10^−4^ mm^2^]: Pre, 19.7, 18.1; UCCAO, 18.7, 26.2; BCCAO, 12.4, 24.2; p = 0.4219) in LS group showed no significant difference among all three phases.

On the first side in LS group, ADC_K_ at the lateral part (n = 4, median, IQR [×10^−4^ mm^2^]: Pre, 7.50, 0.50; UCCAO, 7.08, 0.41; BCCAO, 7.17, 0.25; p = 0.00019, Friedman test) and F_K_D*_K_ at the internal part (Pre, 23.3, 18.8; UCCAO, 17.5, 19.9; BCCAO, 10.0, 12.3; p = 0.00659) showed the significant difference among all phases, and between Pre and UCCAO or BCCAO (p < 0.05, Conover *post hoc* test), while no significant differences were observed those parameters at the same parts in non-LS group (ADC_K_: Pre, 7.33, 0.37; UCCAO, 7.83, 0.84; BCCAO, 7.17, 0.42; p = 0.4979; F_K_D_K_: Pre, 35.1, 30.5; UCCAO, 28.7, 22.7; BCCAO, 8.43, 15.0; p = 0.2687). And then, at the medial part on this side, ADC_K_ showed the significant difference in both groups among all phases (LS: Pre, 8.25, 0.33; UCCAO, 8.00, 0.16; BCCAO, 8.00, 0.16; p = 0.0240; non-LS: Pre, 8.17, 0.42; UCCAO, 8.33, 0.54; BCCAO, 7.50, 0.29; p = 0.0168). Interestingly, at the part, ADC_K_ in LS group significantly decreased between Pre and UCCAO, or BCCAO phases, however, ADC_K_ in non-LS group significantly decreased between UCCAO and BCCAO phases (p < 0.05, Conover *post hoc* test).

On the second side, ADC_K_ at the internal part showed the significance in only non-LS group (Pre, 8.17, 1.16; UCCAO, 7.83, 0.91; BCCAO, 7.33, 0.41; p = 0.02560) but LS group (Pre, 7.83, 0.42; UCCAO, 7.58, 0.33; BCCAO, 7.25, 0.58; p = 0.08374). And, in non-LS group, there was the significant difference in ADC_K_ at the part between UCCAO and BCCAO (p < 0.05, Conover *post hoc* test). No significant difference among all phases was identified at lateral parts on the second side in both groups (LS: Pre, 8.08, 0.33; UCCAO, 7.92, 0.25; BCCAO, 7.50, 0.66; p = 0.09832; non-LS: Pre, 8.17, 0.58; UCCAO, 8.00, 1.17; BCCAO, 7.50, 0.54; p = 0.12074).

## Discussion

The results of this study demonstrated that the features of the IVIM parameters change with depending on the DWI signal model used for the parameter calculation; in particular, the parameter determined with the Kur model identified the largest number of regions showing the significant change in the rat cortex among three CCAO phases in the three examined models. AIC value, which represents the good of fitness between experimental and simulated signals, was also the smallest with Kur model in the models. These results suggested that Kur model may have the highest sensitivity for detecting the ischemic damage in three models. Then, IVIM analysis with all models demonstrated that the cortex on the second ligation side were damaged more severe than that on the first ligation side. This tendency was also observed on MRA. Actually, blood flow reduction on MRA was identified at UCCAO on the second ligation side, which indicates the second ligation side might have been already damaged in UCCAO phase. Furthermore, in a subgroup analysis according to the survival period of the animals, the flow reduction on MRA recovered at BCCAO in LS group; however, the flow reduction remained in non-LS group. Finally, ADC_K_ in non-LS group significantly reduced between UCCAO and BCCAO, while no reduction on ADC_K_ was observed between the two phases in LS group. These suggested that damage due to the flow reduction on the second side in BCCAO phase may affect the survival period for rats. The high sensitivity due to the high signal-to-noise ratio in 11.7TMRI may also be helpful to identify subtle differences in abnormal cerebral perfusion in rats exposed to CCAO. No previous study has quantified ischemic changes in the rat cortex after CCAO using IVIM parameters while simultaneously assessing the cerebral ischemia using preclinical 11.7TMRI *in vivo*.

For many years, IVIM parameters have been mainly used to analyze abdominal regions in clinical studies distinguishing between benign and malignant tumors^14–16,30,31^. The primary benefit of IVIM is its ability to detect lesions in patients without the need for contrast agents. Despite this prominent advantage, only a few reports with cerebral perfusion measurements using IVIM have been published^8,9^. This may be the reason why radioactive nuclide-based imaging modalities like positron emission tomography or single-photon emission computed tomography with their high sensitivity were preferentially used to detect abnormal vascularity and/or perfusion when the magnetic field strength was still lower than 3T. A recent study demonstrated changes in CBF after UCCAO and BCCAO using three-dimensional arterial spin labeling (3D-ASL)^27^. However, 3D-ASL experiences difficulties to accurately assess hemodynamic impairments because of labeling failures due to susceptibility artifacts at the extracranial portion of the ICA or the delayed arrival of labeled blood in patients with chronic ischemia due to atherosclerotic ICA stenosis. IVIM parameters can non-invasively and simultaneously determine brain damage with the parameter ADC, as well as cerebral blood flow (CBF), using just one dataset acquired by a conventional DWI sequence with multiple b-values. Thus, we believe that IVIM can help to identify abnormalities in patients with chronic ischemia showing subtle changes in cerebral blood flow and metabolism, similar to its application in acute stroke as demonstrated by previous publications^8,9^.

In previous studies in rats, tortuosity or dilation of blood vessels have been surgically validated in the chronic phase after BCCAO^2,27,32^. In the present study, such morphological changes in the BA and the ACA were also identified *in vivo* using 11.7TMRI in three different occlusion phases. It is well-documented that autoregulation of blood vessels plays an important role in maintaining CBF in hemodynamic ischemia or hypoxia to provide sufficient oxygen supply for the cerebral metabolism^33,34^. Thus, the morphological changes in the BA and ACA observed *in vivo* in the present study might be a physiological response to ischemia that tries to maintain the cerebral perfusion pressure by preventing a critical pressure reduction. The significant reduction in the cross-sectional area of the ACA between UCCAO and BCCAO might be an apparent structural change due to flow reduction because this reduction can affect the flow void. Finally, ADC reduction were observed at BCCAO phase in all models in the present study, thus, compensation by the autoregulation may be insufficient for the CBF reduction due to the bilateral occlusion.

In the present study, the Kur model showed the lowest AIC value in the fitting procedure among the three examined DWI models, and this model could detect the significant change of IVIM parameters among the three occlusion phases at more regions than the other two models. DWI signals at high b-values mainly represent signals from diffusion components in biological tissue with multiple cell compartments and/or the extracellular space^5,6,35^, whereas DWI signals at low b-values include more perfusion effects in addition to diffusion components, according to the IVIM theory^5,6^. In the present study, the AIC value of the Tri model was higher than that of the Bi and Kur models. This suggests that it might be difficult for the Tri model to biologically represent ischemic changes in the rat cortex, although this model has the advantage to describe complex structures in biological tissues with three exponential terms. In the present study, the Bi model had an AIC value similar to that of the Kur model; however, the AIC was significantly larger than that of Kur. The Bi model can represent only Gaussian distribution of water molecule movement because of no term representing the non-Gaussian component. On other side, the Kur model includes a term representing diffusion changes with both Gaussian and non-Gaussian distributions^36^. Therefore, our results suggest that it is important to assess the effects of non-Gaussian, as well as Gaussian, diffusion compartments to detect ischemic changes with high sensitivity.

Considering the principles of IVIM, the parameter F is theoretically defined as the factor indicating the volume fraction of the fast diffusion and mainly altered by the volume of perfusion compartments in the biological tissue^5,6,37^. In fact, it has been reported that F values are elevated due to increased cerebral blood volume (CBV) in healthy subjects with hypercapnia or in patients with tumors of high vascularity^15,16,19–21,31^. Unfortunately, our results could not identify significant increases in F values among the three examined occlusion phases. Morphological changes of large vessels like the BA, ACA and ICA are qualitatively and quantitatively confirmed in the present study; however, CBV elevation is caused by dilation of precapillary resistance vessels^33^. We hypothesize that our time point for the MRI scan might have missed the period with vasodilation of the small vessels in the cortex. Because this period with vasodilation may occur immediately after CCAO surgery and might be very short, we could not identify elevated F values that indicate an increased blood volume in the rat cerebral cortex.

The results of the subgroup analysis demonstrated hemodynamic and ischemic differences between two rat groups defined by different mortality after CCAO. Interestingly, in the UCCAO phase, a blood flow reduction in the circle of Willis portion of the ICA was identified by MRA only on the side contralateral to the occlusion in both groups. This contralateral flow reduction might indicate that the blood flow compensated for the ligation-sided shortage in blood supply and led to a ‘steal phenomenon’ similar to that observed by single-photon emission computed tomography imaging after intravenous acetazolamide injection in patients with dysfunction of the cerebrovascular autoregulation due to chronic ischemia^38^. On the other hand, only rats in the LS group exhibited recovery of the MRA-assessed blood flow in the BCCAO phase but not those in the non-LS group. The CBF reduction between UCCAO and BCCAO might be more severe in non-LS group than LS group because ADC_K_ significantly reduced between the two phases in only this group but LS group.

This study has some limitations. First, the sample size was small for group comparisons because the rat model had high mortality rates even if the staged ligation was successfully performed. Second, we performed no sham operations. A previous study identified no signs of degeneration or changes in vascularity over 12 weeks in sham-operated rats as control animals to BCCAO rats^2^, but further studies are needed to confirm that the surgical procedures do not affect IVIM parameters in sham-operated rats. Third, we could not directly confirm the relationship between IVIM parameters and the actual cerebral blood flow in CCAO rats using simultaneously other sequences like 3D-ASL^27,38^.

In conclusion, we demonstrated IVIM parameters determined in ultra-high-field 11.7TMRI changed in the rat cortex after CCAO. In particular, the parameters calculated by the kurtosis model could represent ischemic changes in rats with higher fitting accuracy and higher sensitivity compared to the other two models. IVIM parameters determined with suitable DWI signal models may help us to accurately identify subtle ischemic changes in patients as well as in animal models, although we still require the further investigation with larger sample size.

## Methods

### Animals

All experimental procedures involving animals and their care were carried out in accordance with the Guidelines of Osaka University for Animal Experimentation and the National Institutes of Health Guide for the Care and Use of Laboratory Animals. All experimental protocols were approved by the Research Ethics Committee of Osaka University. This manuscript follows the ARRIVE guidelines (Animal Research: Reporting *in Vivo* Experiments). Rats were acclimatized to the facility for seven days before the operation. A total of 27 Wistar rats were used for this study. Preliminary experiments were performed on 17 rats with different intervals of ligation (from zero to six days) to determine a suitable interval between the first and the second CCA ligation to keep the rats longer alive using techniques proposed in previous studies^27,39^. The period of six days between the first and the second ligation was selected owing to the low mortality involved and the decrease in anatomical and/or functional laterality due to the development of new arterial circulations facilitating collateral flow^40^. After this preliminary phase, we performed the following surgical procedures on 11 female Wistar rats (eight weeks old; body weight, 156.5 ± 7.1 g) including one rat treated with the six-day-interval in the preliminary experiment: Following a permanent occlusion of the right CCA with a 4–0 surgical thread ligation, the left CCA was occluded six days after this unilateral occlusion. All surgical procedures were performed under general anesthesia with 2.00–2.54% isoflurane (Abbott Laboratories, Abbott Park, IL, USA) mixed with room air at a flow rate of 2 L/min. All rats were housed in cages under controlled temperature (20–22 °C) and humidity (50–55%) after the surgical procedures. Rats were carefully observed during this study to prevent malnutrition due to anosmia and blindness immediately after the BCCAO, but all animals had *ad libitum* access to food and water during the follow-up period. Rats that survived for two weeks after the BCCAO were assigned to the LS group, whereas the other rats were assigned to the non-LS group.

### MRI

IVIM-DWI (multi-shot spin-echo echo-planar imaging sequence; repetition time (TR)/echo time (TE): 4500/23.1 [ms]; matrix: 64 × 128; field of view (FOV): 12.8 × 25.6 [mm^2^]; in-plane resolution: 0.2 × 0.2 [mm^2^]; slice thickness: 0.8 [mm]; slice gap: 0.2 [mm]; 12 b-values: 0, 10, 20, 40, 80, 160, 320, 640, 800, 1000, 2000, 3000 [s/mm^2^]; diffusion gradient duration time (δ)/diffusion gradient separation time (Δ): 5/13 [ms]; motion probing gradient: three orthogonal directions) was performed in a vertical-bore preclinical 11.7 T MRI scanner (gradient strength: 750 [mT/m], slew rate: 6660 [T/m/s]; AVANCE II 500WB, Bruker) with a transmit/receive volume coil. The scan phase comprised a baseline scan (Pre) followed by a scan three days after the first surgical procedure on the right CCA and a scan within one hour after the second procedure on the left CCA. For assessment of anatomical structures, two-dimensional time of flight (TR/TE: 20/40.8 [ms]; echo train: 16; matrix: 256 × 256; FOV: 25.6 × 25.6 [mm^2^]; in-plane resolution: 0.1 × 0.1 [mm^2^]; slice thickness: 0.3 [mm] without gaps) MRA was performed, and a RARE sequence (TR/effective TE: 6500/38 [ms]; matrix: 256 × 256; FOV: 25.6 × 25.6 [mm^2^]; in-plane resolution: 0.1 × 0.1 [mm^2^]; slice thickness: 0.5 [mm] without gaps) was used to obtain T2-weighted images. Rats were placed on an MRI-compatible cradle and restrained with a bite bar during scanning. MRI scanning for all rats was performed under anesthesia with a 1.50–2.04% isoflurane air mixture at a flow rate of 2 L/min. Respiratory signals were monitored using a physiological monitoring system (SA Instruments, Inc., Stony Brook, NY, USA). Warming pads with circulating water were used to maintain body temperature.

### Assessment of the cerebrovascular morphological and flow changes after CCAO

As reported in previous studies, cerebrovascular morphological changes can be identified in rats after CCAO^2,27,32^. To confirm the effects on the vessels due to severe ischemia, Dia__BA_ and Area__ACA_ were measured in each rat at Pre, UCCAO, and BCCAO. To measure Dia__BA_, we generated an image reformatted along the centerline of the BA using a curved planar reformation technique from axial RARE images. The length of a straight line orthogonally across the centerline of the vessel at the middle of the BA was defined as Dia__BA_. The average of the bilateral cross-sectional ACA areas measured at an axial section image showing the anterior commissure on a RARE image was defined as Area__ACA_. For assessment of the cerebral flow reduction after CCAO, the cross-sectional area of the ICA flow in the circle of Willis on an MRA was measured in each rat similar to the Area__ACA_ measurement on a RARE image. All measurements of cerebrovascular morphological parameters were manually performed twice using OsiriX Lite ver. 8.5 (Pixmeo, Bernex, Switzerland).

### Assessment of cerebral ischemic states by IVIM parameters

To quantitatively assess cerebral hemodynamic changes, each IVIM parameter set was estimated using averaged DWI signals in each ROI on the cortex of each rat. ROIs were automatically placed at the internal, medial, and lateral parts on both right and left sides of the cortex, and DWI signals averaged in each ROI over the four slices, which covered areas mainly perfused by the anterior and middle cerebral arteries, were used for the IVIM parameter estimation. ROI locations were automatically determined by an in-house software as follows: First, the rat brain region was roughly discriminated from the background in each image slice scanned with b = 1000 s/mm^2^ by thresholding; second, the edge line of the cortex was determined by an edge-detection algorithm using an active contour, and the restricted region within this contour was defined as the rat brain region; third, an image erosion was performed to the brain region to determine 4-pixel depth from the brain surface; fourth, the gravity of the eroded image region was calculated, and the horizontal line through the gravity was defined on the image; and fifth, the line from the lateral edge to the gravity on the horizontal line was divided into five sections, and the positions at 1/5, 2/5, and 3/5 from the gravity on the horizontal line were defined as the internal, medial, and lateral position on the horizontal direction, respectively. Finally, a ROI with 2-pixel radius was placed on each position; thus, six ROIs were placed in each rat (Fig. 3).

We used the following three typical diffusion-weighted signal models^5,6^ for all IVIM parameter estimations:

Bi-exponential model (Bi)

1 $${\rm{S}}={S}_{B0}\{{F}_{B}\exp (-b{{D}_{B}}^{\ast })+(1-{F}_{B})\exp (-bAD{C}_{B})\}$$

Kurtosis model (Kur)

2 $${\rm{S}}={S}_{K0}\{{F}_{K}\exp (-b{{D}_{K}}^{\ast })+(1-{F}_{K})\exp \{-bAD{C}_{K}+K{(bAD{C}_{K})}^{2}/6\}\}$$

Tri-exponential model (Tri)

3 $${\rm{S}}={S}_{T0}\{{F}_{T}\exp (\,-\,b{{D}_{T}}^{\ast })+(1-{F}_{T})\{{f}_{s}\exp (-b{D}_{s})+(1-{f}_{s})\exp (-b{D}_{f})\}\}$$

(S_0_, the signal on the image acquired with b = 0; F, the fractional volume of capillary blood flowing; D* [mm^2^/s], the pseudo diffusion coefficient correlated with blood flow velocity; ADC [mm^2^/s], the apparent diffusion coefficient in the tissue; K, the coefficient for restricted diffusion weighting; f_s_, the fractional volume of the slow diffusion compartment; D_s_ and D_f_ [mm^2^/s], ADC in the slow and diffusion compartment, respectively). In addition, we calculated FD* as a parameter correlated with the CBF, as well as an ADC value for the Tri model ADC_T_ = f_s_D_s_ + (1 − f_s_)D_f_ for comparison with ADC_B_ and ADC_K_^6^. Subsequently, all IVIM parameter sets in the three models were estimated using a range-restricted exhaustive search method with the DWI signal database, without curve fitting procedures as approved in a previous study^41^. This method is advantageous as it does not show divergence in the parameter estimation procedure, as observed in general curve fitting procedures. The range-restricted exhaustive search method can automatically decide the optimal IVIM parameters in each formula as follows: First, a simulative DWI signal dataset with data points having the same number as b-values was calculated using the formula with the combination of IVIM parameters, of which a suitable range and steps for the rat cortex were previously defined; second, a DWI signal database was established with all simulative DWI signal datasets calculated by all combinations of IVIM parameters; third, the mean square errors (MSEs) between each simulated DWI signal dataset in the database and an experimental DWI dataset from a rat were calculated; and fourth, a combination of IVIM parameters simulating the best DWI signal dataset showing the minimum MSE was defined as the optimal combination of IVIM parameters. In this study, all experimental DWI signals were normalized to 1000 at b = 10 [s/mm^2^], and a DWI signal database was experimentally established with the following ranges and steps covering the range of common IVIM/DWI parameters of the brain as described in previous publications^7–10,13,23,41^: [minimum, maximum: step] (unit); S_0_: [1000, 1040: 10]; F: [0, 0.20: 0.01]; D*: [0.002, 20.0: 0.2] (×10^−2^ mm^2^/s); ADC: [0.0, 20.0: 1.0] (×10^−4^ mm^2^/s); K: [0.0, 1.2: 0.1]; f_s_: [0.0, 0.5: 0.05]; D_s_: [0.0, 5.0: 0.2] (×10^−4^ mm^2^/s); and D_f_: [5.0, 20.0: 4.0] (×10^−4^ mm^2^/s). Finally, the numbers of parameter combinations that equal the numbers of simulated DWI signal datasets establishing the database are 220500, 2866500, and 12012000 for the Bi, Kur, and Tri models, respectively.

As an index to confirm the degree of the adaptation of each model in the IVIM parameter estimation, AIC considering a small sample size estimation was calculated using the MSE according to the following formula^10,28,29^:

4 $${\rm{AIC}}={N}_{b}\,\mathrm{ln}(MSE)+\frac{2k(k+1)}{{N}_{b}-k-1}$$

(N_b_, number of b-values used for the parameter estimation; k, number of parameters in the model). A small AIC value implies that the adaptation of the model to the experimental signals is good. All calculation procedures were automatically performed using our custom-made software in MATLAB (Mathworks, Natick, MA, USA).

### Statistical analysis

Significant differences in Dia__BA_, Area__ACA_, ICA flow or IVIM parameters were examined among the three phases (Pre, UCCAO, and BCCAO) using the Friedman test with the Conover *post hoc* test. The accuracy of the two measurements for Dia__BA_, Area__ACA_ or ICA flow was assessed using intraclass correlation coefficients, which can validate the reliability of the measurement in an operator. Significant differences in IVIM parameters were examined among the three phases for non-LS or LS groups using the Friedman test with pairwise comparisons by the Conover *post hoc* test. Significant differences in AICs for all estimations were examined among the three models using the repeated measures analysis of variance with pairwise comparisons using the significant criteria corrected by Bonferroni correction. All statistical analyses were performed on MedCalc ver. 17.9.7 (MedCalc Software bvba, Ostend, Belgium) with a significance level of p < 0.05.

## Supplementary information

Supplementary information.
