# Supplementary material for: Feasibility of IVIM parameters from diffusion-weighted imaging at 11.7T MRI for detecting ischemic changes in common carotid artery occlusion rats
Source: Sci Rep. 2020 May 21;10:8404. doi: 10.1038/s41598-020-65310-8 (PMC7242437; doi:10.1038/s41598-020-65310-8)
Supplement: Supplementary file 1 — Supplementary information. [file 41598_2020_65310_MOESM1_ESM.pdf]

Feasibility of IVIM parameters from diffusion-weighted imaging at 11.7T MRI for detecting ischemic changes in common carotid artery occlusion rats

Shunrou Fujiwara[1,2], Yuki Mori[3], Daniela Martinez de la Mora[2], Yosuke Akamatsu[1], Kenji Yoshida[1]
Yuji Shibata[4], Tomoyuki Masuda[4], Kuniaki Ogasawara[1], Yoshichika Yoshioka[2,5]

[1]Department of Neurosurgery, Iwate Medical University, 1-1-1 Idaidori, Yahaba, Iwate 028-3694, Japan
[2]Graduate School of Frontier Science, Osaka University, 3-1 Yamadaoka, Suita, Osaka 565-0871, Japan
[3]Center for Translational Neuromedicine, University of Copenhagen, Blegdamsvej 3B, 2200 Copenhagen N, Denmark
[4]Department of Pathology, Iwate Medical University, 1-1-1 Idaidori, Yahaba, Iwate 028-3694, Japan
[5]Center for Information and Neural Networks (CiNet), NICT and Osaka University, 3-1 Yamadaoka, Suita, Osaka 565-0871, Japan

Results at the lateral part of the left cortex.

	Pre		UCCAO		BCCAO		
	Median	25-75% percentile	Median	25-75% percentile	Median	25-75% percentile	p value
F_B (%)	15.50	13.70 – 16.00	15.50	13.90 – 16.00	15.20	12.60 – 16.00	0.4853
D_B* [$\times10^{-3}$ mm^2/s]	4.00	3.58 – 7.08	3.67	3.33 – 30.00	5.67	3.00 – 9.25	0.7394
F_BD_B* [$\times10^{-4}$ mm^2/s]	6.27	5.27 – 9.67	5.68	5.36 – 45.20	7.46	5.09 – 11.8	0.9054
ADC_B [$\times10^{-4}$ mm^2/s]	6.50	6.33 – 6.67	6.33	6.17 – 6.75	6.00*, †	5.96 – 6.50	0.0062
F_K (%)	6.83	5.13 – 7.96	5.67	4.88 – 8.96	7.50	5.67 – 9.50	0.6669
D_K* [$\times10^{-3}$ mm^2/s]	26.30	16.40 –51.50	40.00	8.17 – 44.50	8.33	5.92 – 33.5	0.0906
F_KD_K* [$\times10^{-4}$ mm^2/s]	24.60	9.12 – 28.30	22.70	6.48 – 27.70	7.60	2.75 – 27.3	0.4853
ADC_K [$\times10^{-4}$ mm^2/s]	8.17	7.83 – 8.21	8.00	7.79 – 8.17	7.50*, †	7.17 – 7.83	0.0095
F_T (%)	6.50	3.71 – 7.08	4.83	4.54 – 6.83	7.17	3.29 – 8.00	0.3680
D_T* [$\times10^{-3}$ mm^2/s]	42.70	24.20 – 52.3	39.30	8.58 – 49.6	15.70	11.60 – 31.60	0.1019
F_TD_T* [$\times10^{-4}$ mm^2/s]	30.30	7.40 – 35.0	20.20	5.96 – 29.3	10.40	5.39 – 16.60	0.2467
ADC_T [$\times10^{-4}$ mm^2/s]	8.39	7.83 – 8.69	8.12	7.76 – 8.36	7.56*	7.23 – 7.90	0.0211

*, significance compared to Pre with p<0.05
†, significance compared to UCCAO with p<0.05

Results at the medial part of the left cortex.

	Pre		UCCAO		BCCAO		
	Median	25-75% percentile	Median	25-75% percentile	Median	25-75% percentile	p value
F_B (%)	15.70	14.20 – 17.00	14.00	12.90 – 16.00	14.50	13.50 – 16.40	0.9749
D_B* [$\times 10^{-3}$ mm^2/s]	4.00	2.92 – 13.8	7.33	3.58 – 37.1	3.67	32.5 – 50.8	0.2773
$F_B D_B$* [$\times 10^{-4}$ mm^2/s]	6.67	4.80 – 21.40	10.40	5.27 – 46.4	5.56	5.05 – 6.64	0.2467
ADC_B [$\times 10^{-4}$ mm^2/s]	6.50	6.25 – 6.83	6.33	6.17 – 6.71	6.17	6.00 – 6.37	0.0990
F_K (%)	6.50	5.79 – 7.67	6.17	5.38 – 6.87	6.17	5.38 – 7.92	0.4853
D_K* [$\times 10^{-3}$ mm^2/s]	40.30	20.20 – 49.30	37.70	10.60 – 63.40	18.70	10.80 – 38.30	0.4853
$F_K D_K$* [$\times 10^{-4}$ mm^2/s]	21.20	7.23 – 38.10	20.20	7.23 – 38.10	11.80	7.55 – 18.70	0.2467
ADC_K [$\times 10^{-4}$ mm^2/s]	8.00	7.83 – 8.08	7.83	7.67 – 8.17	7.67	7.33 – 7.87	0.1485
F_T (%)	5.67	4.88 – 7.83	6.67	5.83 – 7.83	5.00	3.63 –6.46	0.1720
D_T* [$\times 10^{-3}$ mm^2/s]	40.30	32.70 – 56.10	41.00	7.92 – 56.9	19.00	10.70 – 39.30	0.7394
$F_T D_T$* [$\times 10^{-4}$ mm^2/s]	23.90	18.40 – 30.00	27.90	6.20 – 31.80	9.50	5.07 – 16.80	0.3897
ADC_T [$\times 10^{-4}$ mm^2/s]	8.00	7.59 – 8.28	7.62	7.49 – 7.98	7.63	7.50 – 7.90	0.4853

Results at the internal part of the left cortex.

	Pre		UCCAO		BCCAO		
	Median	25-75% percentile	Median	25-75% percentile	Median	25-75% percentile	p value
F_B (%)	15.80	13.50 –17.0	14.80	13.20 – 15.50	13.70	13.50 – 15.7	0.6079
D_B* [$\times10^{-3}$ mm^2/s]	4.00	3.17 – 11.5	4.00	3.33 – 22.5	4.67	3.42 – 5.50	0.7138
F_BD_B* [$\times10^{-4}$ mm^2/s]	6.13	5.09 – 15.00	5.50	4.79 – 32.8	6.10	5.12 – 8.01	0.7394
ADC_B [$\times10^{-4}$ mm^2/s]	6.33	6.33 – 6.54	6.33	6.29 – 6.58	6.00*, †	5.96 – 6.21	0.0004
F_K (%)	7.17	5.42 – 8.58	7.00	6.17 – 7.54	7.00	6.25 – 9.42	1.0000
D_K* [$\times10^{-3}$ mm^2/s]	16.30	8.08 – 42.80	9.33	4.83 – 56.9	10.00	5.83 – 27.5	1.0000
F_KD_K* [$\times10^{-4}$ mm^2/s]	13.30	4.70 – 29.50	6.53	2.66 – 37.20	7.23	4.38 – 23.7	0.4853
ADC_K [$\times10^{-4}$ mm^2/s]	7.83	7.58 – 8.33	7.67	7.63 – 8.08	7.33*, †	7.08 – 7.33	0.0066
F_T (%)	7.83	4.54 – 8.33	6.17	5.12 – 7.00	5.83	4.38 –6.25	0.7930
D_T* [$\times10^{-3}$ mm^2/s]	21.00	7.50 – 53.60	45.00	7.25 – 59.1	12.30	7.25 – 39.3	0.4853
F_TD_T* [$\times10^{-4}$ mm^2/s]	6.13	5.09 – 15.00	5.50	4.79 – 32.8	6.10	5.12 – 8.01	0.7394
ADC_T [$\times10^{-4}$ mm^2/s]	8.13	7.29 – 8.35	7.79	7.61 – 8.00	7.43*, †	7.25 – 7.55	0.0336

*, significance compared to Pre with $p<0.05$

†, significance compared to UCCAO with $p<0.05$

Results at the internal part of the right cortex.

	Pre		UCCAO		BCCAO		
	Median	25-75% percentile	Median	25-75% percentile	Median	25-75% percentile	p value
F_B (%)	14.80	14.20 –15.20	14.70	13.40 – 17.00	13.30	12.20 – 16.30	0.4853
D_B* [$\times 10^{-3}$ mm^2/s]	3.20	2.62 – 3.53	2.73	2.18 – 2.95	2.77	2.13 – 2.97	0.2773
F_BD_B* [$\times 10^{-4}$ mm^2/s]	8.09	5.45 – 18.7	10.50	4.59 – 38.8	6.79	5.18 – 9.17	0.6669
ADC_B [$\times 10^{-4}$ mm^2/s]	6.33	6.17 – 6.67	6.17	6.04 – 6.50	6.17	5.83 – 6.37	0.5578
F_K (%)	7.17	6.38 – 8.92	7.83	6.54 – 8.46	5.67	4.75 – 7.12	0.2476
D_K* [$\times 10^{-3}$ mm^2/s]	33.30	15.60 – 53.00	35.70	16.60 – 49.70	21.00	11.00 – 38.30	0.1178
F_KD_K* [$\times 10^{-4}$ mm^2/s]	29.40	13.70 – 35.60	23.60	11.60 – 34.40	9.90*	7.70 – 21.80	0.0211
ADC_K [$\times 10^{-4}$ mm^2/s]	7.67	7.46 – 8.17	7.50	7.46 – 7.92	7.50	7.33 – 7.54	0.4140
F_T (%)	5.33	5.04 – 6.54	5.33	4.71 – 6.92	5.83	3.50 –6.42	0.9054
D_T* [$\times 10^{-3}$ mm^2/s]	47.70	30.00 – 60.40	45.70	28.80 – 70.80	20.70	16.80 – 43.40	0.2467
F_TD_T* [$\times 10^{-4}$ mm^2/s]	25.10	12.90 – 32.70	30.90	13.40 – 35.60	13.10	7.66 – 20.50	0.1178
ADC_T [$\times 10^{-4}$ mm^2/s]	7.97	7.72 – 8.47	7.81	7.46 – 8.05	7.40	7.36 – 7.55	0.2773

*, significance compared to Pre with $p<0.05$

Results at the medial part of the right cortex.

	Pre		UCCAO		BCCAO		
	Median	25-75% percentile	Median	25-75% percentile	Median	25-75% percentile	p value
F_B (%)	15.30	14.10 – 16.10	13.70	13.00 – 15.90	15.00	14.50 – 16.20	0.4641
D_B* [$\times 10^{-3}$ mm^2/s]	5.00	4.33 – 10.4	4.00	3.58 – 21.4	4.67	3.83 – 5.83	0.8310
$F_B D_B$* [$\times 10^{-4}$ mm^2/s]	7.16	6.31 – 16.90	6.07	5.50 – 31.1	6.77	5.91 – 9.19	1.0000
ADC_B [$\times 10^{-4}$ mm^2/s]	6.50	6.46 – 671	6.67	6.46 – 6.92	6.17*, †	5.96 – 6.54	0.0001
F_K (%)	7.17	6.00 – 7.88	6.50	5.88 – 7.79	7.17	3.83 – 10.2	0.9263
D_K* [$\times 10^{-3}$ mm^2/s]	31.70	17.70 – 41.30	40.30	11.0 – 49.50	13.70	7.42 – 43.40	0.3897
$F_K D_K$* [$\times 10^{-4}$ mm^2/s]	23.80	9.72 – 30.40	24.30	11.0 – 31.20	9.07	6.19 – 20.40	0.2586
ADC_K [$\times 10^{-4}$ mm^2/s]	8.17	8.17 –8.42	8.17	7.96 – 8.33	7.67*	7.46 – 8.00	0.0495
F_T (%)	6.67	5.04 – 8.75	7.67	6.25 – 9.50	4.67	3.96 –7.92	0.7394
D_T* [$\times 10^{-3}$ mm^2/s]	35.00	18.80 – 57.80	41.70	27.5 – 49.5	38.30	13.20 – 43.90	0.7394
$F_T D_T$* [$\times 10^{-4}$ mm^2/s]	27.20	13.40 – 33.90	22.90	18.00 – 39.90	15.60	6.62 – 21.20	0.4853
ADC_T [$\times 10^{-4}$ mm^2/s]	8.39	7.88 – 8.58	8.12	7.53 – 8.42	7.78	7.60 – 8.11	0.4853

*, significance compared to Pre with p<0.05
†, significance compared to UCCAO with p<0.05

Results at the lateral part of the right cortex.

	Pre		UCCAO		BCCAO		
	Median	25-75% percentile	Median	25-75% percentile	Median	25-75% percentile	p value
F_B (%)	15.60	14.50 – 15.80	14.60	13.70 – 15.60	14.70	13.80 – 15.40	0.2467
D_B* [$\times 10^{-3}$ mm^2/s]	5.83	3.88 – 13.10	5.94	3.35 – 31.10	4.33	3.67 – 8.75	0.4853
$F_B D_B$* [$\times 10^{-4}$ mm^2/s]	8.48	5.88 – 16.70	8.26	5.15 – 46.20	6.19	5.71 – 11.8	0.2467
ADC_B [$\times 10^{-4}$ mm^2/s]	6.36	6.24 – 6.61	6.33	6.18 – 6.58	6.03*, †	5.92 – 6.27	0.0335
F_K (%)	6.86	6.60 – 7.44	6.69	6.53 – 7.30	6.94	5.75 – 7.56	0.8355
D_K* [$\times 10^{-3}$ mm^2/s]	35.80	16.70 – 44.50	25.50	16.9 – 51.00	19.20*, †	12.90 – 32.00	0.0335
$F_K D_K$* [$\times 10^{-4}$ mm^2/s]	27.10	9.65 – 31.00	15.70	11.2 – 34.50	13.80*, †	7.84 – 17.50	0.0335
ADC_K [$\times 10^{-4}$ mm^2/s]	7.89	7.72 – 8.06	7.72	7.57 – 8.03	7.47*, †	7.35 – 7.50	0.0054
F_T (%)	6.33	4.66 – 7.29	6.17	5.49 – 6.94	5.14	4.31 – 6.98	0.6669
D_T* [$\times 10^{-3}$ mm^2/s]	38.10	25.30 – 50.2	47.10	23.8 – 58.5	22.10†	14.80 – 44.70	0.0091
$F_T D_T$* [$\times 10^{-4}$ mm^2/s]	22.90	12.00 – 31.30	23.40	13.4 – 38.20	14.30†	6.76 – 23.20	0.0211
ADC_T [$\times 10^{-4}$ mm^2/s]	8.16	7.62 – 8.44	7.72	7.56 – 7.96	7.50	7.34 – 7.59	0.1178

*, significance compared to Pre with $p<0.05$

†, significance compared to UCCAO with $p<0.05$
